# Assessment of Damage in Composite Pressure Vessels Using Guided Waves

**DOI:** 10.3390/s22145182

**Published:** 2022-07-11

**Authors:** Vittorio Memmolo, Leandro Maio, Fabrizio Ricci

**Affiliations:** Department of Industrial Engineering, University of Naples “Federico II”, Via Claudio 21, 80125 Naples, Italy; leandro.maio@unina.it (L.M.); fabricci@unina.it (F.R.)

**Keywords:** damage detection, Lamb waves, aerospace structures, ultrasound, smart structures

## Abstract

This paper deals with guided wave-based structural health monitoring of composite overwrapped pressure vessels adopted for space application. Indeed, they are well suited for this scope thanks to their improved performance compared with metallic tanks. However, they are characterized by a complex damage mechanics and suffer from impact induced damage, e.g., due to space debris. After reviewing the limited progress in this specific application, the paper thoroughly covers all the steps needed to design and verify guided wave structural health monitoring system, including methodology, digital modelling, reliability, and noise estimation for a correct decision-making process in a virtual environment. In particular, propagation characteristics of the fundamental anti-symmetric mode are derived experimentally on a real specimen to validate a variety of finite element models useful to investigate wave interaction with damage. Different signal processing techniques are demonstrated sensitive to defect and linearly dependent upon damage severity, showing promising reliability. Those features can be implemented in a probability-based diagnostic imaging in order to detect and localized impact induce damage. A multi-parameter approach is achieved by metrics fusion demonstrating increased capability in damage detection with promising implication in enhancing probability of detection.

## 1. Introduction

Composite Overwrapped Pressure Vessels (COPV) have been adopted in recent decades as high-pressure gas containers for different industrial needs, moving from automotive to space applications and passing through energy field for hydrogen storage [1]. A significant COPV design is made by a metal liner, generally aluminium, with a full-composite overwrap, made of a carbon fibre composite which withstands the loads. Usually, composite part is manufactured through filament winding resulting in different plies with different orientation. In addition, a thin epoxy bonding layer is applied to optimally nest the inner metallic hull. The use of carbon fibre composites results in significantly lower weight than all-metal pressure vessels would have. The key benefits of using composites make these storage tanks light-weight, with long-term potential. However, they are likely employed in harsh and unpredictable condition and damage may occur at any time. A maintenance critical damage consists of through thickness composite damage (e.g., crack and delamination) and debonding between metallic and composite parts. In particular, this latter may arise at the interface (decreasing load-carrying capabilities of the structure) due to impact events (e.g., low energy dynamics and space debris). Hence, it is worth integrating a structural health monitoring (SHM) system to warn the presence of a possible detachment which could threaten the component life cycle and dramatically reduce safety.

In this context, SHM is a promising approach conceived in many engineering fields to increase safety and enhance performance of mechanical components and vehicles. The idea behind this approach relies on monitoring characteristic data of the structural component to manage the health status. This can increase safety predicting any unexpected failure which may be due to the harsh environment in which the whole system works. In addition, safety and maintenance requirements of many load carrying structures include scheduled integrity inspections and in-service damage assessment during the whole lifetime. This damage tolerance approach is cost demanding and can be replaced by implementing SHM towards on-condition maintenance, skipping unnecessary inspections and reducing operating costs [2]. Using a cluster of sensors permanently attached to the structure, it is possible to gather information about the load carrying component continuously or on demand [3]. That is to say, on-line and on-demand interrogation of the structure is performed in place of any other inspection task requiring non-destructive testing with a probe to scan the whole structure [4]. The last possibility relies on the use of data warning the presence of every damage during lifetime, which can be derived using several physical knowledge [5]. In addition, the continuous record of such information enable to record the structure aging during lifetime. This allows to warn any overload and eventually estimate the remaining useful life of the structure [6]. Eventually, SHM methodologies can be combined with an integrated sensing system in composite structures, enabling self-sensing capabilities with near-zero mass [7].

Among the various techniques available, electromechanical impedance of the sensors [8], structure vibration response [9,10], and wave propagation in solids [11], are massively used to warn damage because they are affected by structure characteristics. Among them, guided waves (GWs) are well suited to look into many types of shell-like structures, where they can propagate over long distances and interact with emerging flaws. A specific example consists of composite structures, whose media affect wave propagation characteristics and barely visible damage due to low velocity impacts can be detected resorting to a few transducers. In addition, this technique is easily approached for structural health monitoring purpose using light weight piezoelectric transducers enabling full coverage of the structure under inspection. Indeed, continuously monitoring the structure rather than inspecting the component in a non-destructive way can increase safety while reducing operating costs of the vehicle [2,12]. This aspect is even more important for COPVs for space applications because structural component release is not feasible and the non-destructive evaluation should be undertaken during the mission. This is the main reason why SHM approaches have been recently introduced in the space field [13]. However, exploiting ultrasonic waves for condition monitoring is far from simple in such multilayered structures due to the complex propagation behavior. Indeed, Lamb waves are multi-modal and show different defect-mode interaction, requiring a deep knowledge to design an SHM system in order to find out the appropriate wave modes according to the flaw types, and the necessary excitation and suitable sensor configurations [14]. Indeed, a certain defect may arise either in one of the materials or among them. However, according to the specific wave mode adopted, wave energy can be focused mostly in a certain layer, whose damage can be eventually detected appropriately [15]. Otherwise, the scattering behavior can be observed to detect and estimate the size of the damage at the interface [16]. Nonetheless, a step further is needed to move from theoretical wave propagation in multilayered COPV to damage detection. Despite different approaches are available using guided waves in a non-destructive inspection shape [17], there is still a lack of reliable condition monitoring approaches enabling the reliable implementation of a permanently installed SHM system.

From literature review, it looks evident that the use of structural health monitoring for COPV monitoring is not explored thoroughly in the literature. Indeed, the principal papers dealing with this application mostly refer either to the characterization of wave propagation in COPV or to the feasibility of GW modes to interact with damage arising in COPV. In addition, there is not any validation with real pressure vessel components (usually bilayered plates in place of COPV are adopted for the experiments) and no paper deals with the characterization of a whole SHM system devoted to damage detection in COPV.

The results achieved in this paper can be useful qualitatively to every general application in the GW-based SHM field. Although this latter is a wide research framework recent reviews on the topic shows that many aspects are still not addressed thoroughly and can benefit from the presented findings. In particular, focusing on experiments, it is clear that there is still much to improve to reach industrial deployment of such an SHM approach [18]. In this sense, numerical simulations are crucial to help achieving a better knowledge of the physical phenomenon. However, although many numerical approaches have been proposed so far, with their advantages and drawbacks [19], it is still very complex to achieve the validation of a digital physical system and improve SHM approaches introducing a model-based technique.

To close this gap, the present paper covers for the first time all the steps needed to design and verify a GW-based SHM system, including methodology, digital modelling, reliability, and noise estimation for a correct decision-making process in the virtual environment. In this way, this paper investigates the feasibility of guided waves in performing SHM of a COPV in terms of defect detection and localization. Virtual tools are used to support the investigation and idealize the complex structure and simulate wave propagation accurately. To this end, three distinct finite element (FE) models being characterized by different degrees of fidelity are conceived and tested. The comparison of numerical findings with measurements is then carried out to assess the most suited model(s) for SHM simulation. Then, the most suited approach (compared to measurements) is selected to simulate SHM inspection. This latter allows to look into the wave damage interaction and discuss the feasibility of GW-based SHM for COPV. Based on these findings, damage indicators warning the flaw are formulated using disparate signal processing techniques. Finally, a damage reconstruction approach based on probabilistic imaging is implemented to correctly localize the damage.

## 2. Materials and Methods

### 2.1. Statement of the Problem

The COPVs have increasingly been used in many fields as a fluid container. Space structures, launch vehicles, and automotive hollow containers are a few examples of pressure vessels that can be designed using either metallic or composite materials. Generally, pressure vessels are classified in four different categories according to their construction characteristics:

- Type I: all-metal vessel, generally made from steel, is easily produced worldwide and represents the cheapest yet heaviest solution available on the market.
- Type II: mostly steel or aluminum overwrapped by a glass-fiber composite along hoop direction. The metal vessel and composite materials share about equal structural loads, as a result lighter but more expensive than Type I vessels.
- Type III: metal liner, generally aluminum, with carbon fiber composite overwrap, mostly carrying the structural loads.
- Type IV: all-composite vessel made from either carbon or hybrid carbon/glass fiber composite and equipped with a polymer inner liner.

Type III and Type IV COPVs are much lighter than the other vessel categories (constructing pressure vessels, in whole or in part, from composites reduces fuel system and vehicle weight) but require complex manufacturing with specialized technologies to be produced. In addition, they are, as a result, quite expensive and are suitable, either for high value products or in the case of specific needs. Indeed, composite vessels provide better energy storage density, resulting the most practical solution for high-pressure applications. In addition, corrosion resistance and overall safety increases together with the service life, this latter thanks to excellent fatigue resistance shown by composite tows. As a result, they can last up to 30 years before being replaced, which is twice the lifespan of Type I and Type II vessels.

As to the manufacturing issues, it is worth noting that COPV is a multilayered component consisting of a inner liner (typically, aluminum alloy Al6061-T6) and an external composite reinforcement (typically made of carbon fibers) which is wrapped on the metal part. Filament winding technology is employed to manufacture the composite layers and wrap up the cylindrical metal while achieving homogeneous strength of the whole container. The presence of dissimilar materials is accommodated using epoxy adhesive interposed at the metal-composite interface to ensure structural continuity. In this way, any fluid leakage is prevented by the metal liner which contains the pressurized fluid in a sealing manner. Instead, the necessary structural strength is provided by the external wrapping with lower weight possible. Finally, this latter also works as a protection from harsh environment outside. Hence, the composite lay-up is critical maintenance, being both related to load carrier and the barrier between the inner structure and the external environment. Indeed, according to the specific application, the COPV surface is randomly subject to impacts (e.g., space debris, accident induced impact, etc.). Because carbon fiber-reinforced polymers (CFRPs) show very low normal strength along with low interlaminar shear strength between adjacent plies with very different fiber orientation, low velocity impacts may promote damage and delamination can arise between several plies. In addition to that, most likely a debonding between metal and composite site may happen at the interface when the impact energy release breaks the adhesive layer. This consequently decreases the stiffness and crack arising at the interface can propagate making the structure no longer able to withstand loads. This can be threatening and demands for damage tolerance design, including strict conservative rules together with in-service non-destructive inspection. This last schedule is quite demanding, requiring eventually the dismounting of parts, which is particularly unsuitable for space applications because the vehicle cannot be eventually removed from service.

An alternative solution consists of continuous condition monitoring integrating a network of sensors on or within the COPV structure. The idea lies in the design of a permanent structural health monitoring system based on high-frequency diagnostic waves. Ultrasonic transducers are employed to let guided waves propagate through the COPV medium. According to the travelling mode (either symmetric or anti-symmetric), ultrasonic waves show a typical propagation velocity depending upon the excitation frequency and the media properties, which change when damage arises locally. Each transducer can either generate a diagnostic wave (actuator) or record the structural vibration due to the diagnostic input (sensor). Using a actuator–sensor pair, the structural response can be recorded on both pristine and damaged structure and compared in such a way to gather information about the hidden flaw. The idea behind this approach is that using a cluster of sensors, post-processing raw data can return information about damage presence, along with its location and severity.

**Figure 1:**
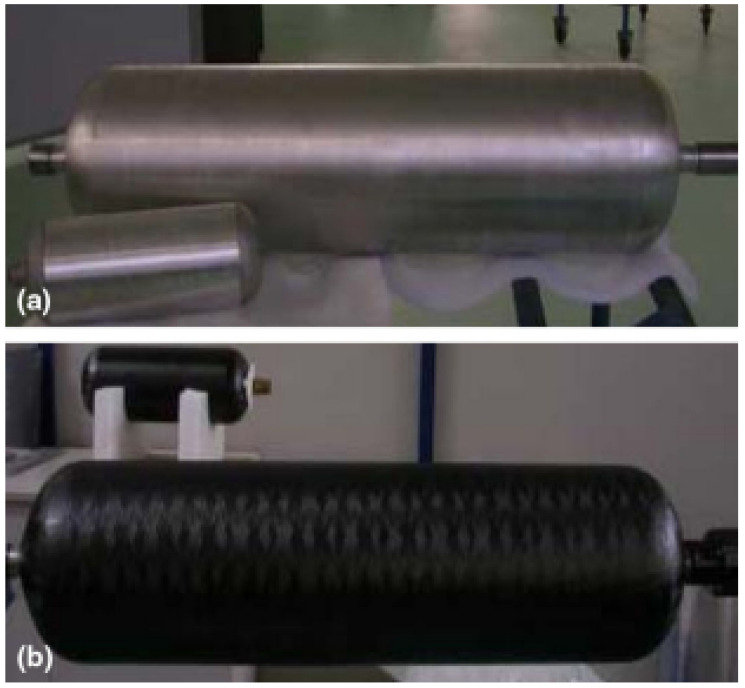
Test article liner (**a**) and the whole structure obtained by filament winding process (**b**) [16].

In this context, a combined experimental and numerical investigation is presented in this paper considering a COPV typically adopted for space application. The full scale of the liner is shown in Figure 1a while the whole container obtained after filament winding is depicted in Figure 1b, respectively. From the pictures’ background a scaled specimen for laboratory tests is also visible. This is the sample adopted for SHM experiments.

In details, the composite layup consists of hoop and helical (unidirectional) plies stacked together resulting in a 3.2-millimeter-thick multilayered composite characterized by the lamination sequence $\left[90_{2}/\pm 11/90_{2}/\pm 11/90_{2}/\pm 11/\pm 11/70_{2}\right]$. Instead, the liner is made from aluminum alloy and is characterized by a 3.35 mm thickness. Finally, the epoxy material interposed at the interface forms a 0.12 mm adhesive layer. Material properties of aluminum alloy, unidirectional composite plies and epoxy adhesive are reported in Table 1 according to the manufacturer specifications. The COPV nominal section results a 170 mm diameter circumference. The cylinder radius is kept to close both tips of the hollow structure.

### 2.2. Experimental Setup

**Figure 2:**
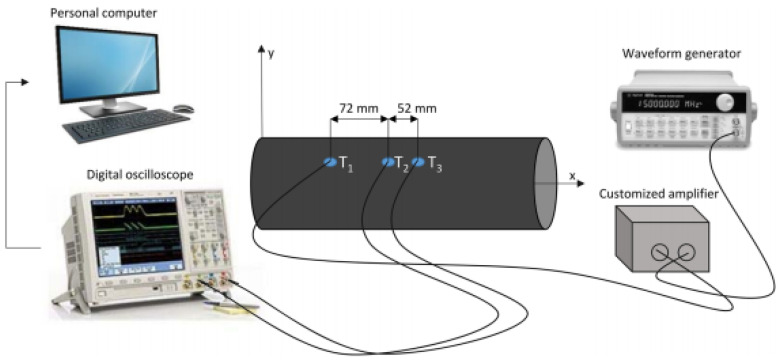
Schematization of the overall experimental setup adopted for wave propagation analysis [16].

Figure 2 shows the experimental setup used to look into the propagation behavior of Lamb waves, whose dispersive characteristics are further exploited to validate numerical simulations.

The laboratory scale COPV shown in Figure 1b is equipped with three lead zirconate titanate (PZT) disks along the longitudinal direction of the container using vacuum-based secondary bonding. The PZT transducer is made of ferroelectric soft piezo material (PIC255) produced by Physik Instrumente and characterized by 10 mm diameter and 0.25 mm thickness. The bonding procedure is used to obtain optimum adhesion between transducer and CFRP, characterized by rough surface. This results in a very thin and homogeneous bonding layer, which practically transmits the deformation of the transducer to the structure through an ultrasonic dry coupled method. The PZT disk returns a time varying strain when excited by a variable voltage load (converse piezoelectric phenomenon). Otherwise, it returns a time dependent voltage when the structure vibrates (direct piezoelectric phenomenon). To this end, disk leads are soldered to electrical cables and connected to either a waveform generator (actuator) or an oscilloscope (sensor). This latter digitizes the signal recorded by the sensor when wave travels across the media. The former (HP/Agilent 33120A) is instead used to generate a tone burst signal to polarize the PZT disk enabling the piezoelectric phenomenon. The diagnostic signal consists of 4.5 sinusoidal cycles windowed by the Hanning function. This waveform is selected to obtain a band bell spectrum (without leakage) tightened around the carrier frequency and characterized by a rather limited duration over time. The frequency spectrum obtained prevents undesired effects due to dispersion while the limited time duration prevents overlapping of waves travelling all around the hollow structure. The signal amplitude is finally boosted up to 80 Vpp through a customized amplifier to obtain a high signal to noise ratio. BNC connectors and twisted and insulated cables are adopted to link the instruments are described before and sketched in Figure 2. Wave propagation is excited in $T_{1}$ in a laboratory with uncontrolled environment in the range 10–80 kHz to characterize the dispersive behavior of the first antisymmetric mode of Lamb waves ($A_{0}$). To this end, the waves passing across $T_{2}$ and $T_{3}$ are recorded and digitized by the oscilloscope to be eventually downloaded on a personal computer. Post-processing ultrasonic data, the time of arrival is then estimated at each frequency using the short-time Fourier transform [20] and the velocity calculated as:

(1) $$v_{g}=\frac{\delta s}{t_{3}-t_{2}}$$

where δs is the distance between the sensors and $t_{i}$ is the time of arrival of the wave at the i-th sensor. This approach is used to avoid undesired transient effect due to the transducer and reduce the error in the velocity estimation [21].

### 2.3. Numerical Simulation

Simulation of wave propagation has been addressed by many authors so far [19]. Among various techniques available, finite element modelling is a flexible and reliable solution as it is implemented in many commercial software with easy pre- and post-processing capabilities. For the scope of this research, FE modelling is implemented in Abaqus virtual environment using transient dynamic analysis to formulate the equations of motion. Meanwhile, the dynamic explicit technique is used to integrate them in order to achieve a fast computing. Indeed, simulating wave propagation in multilayered media requires a very fine discretization which makes the implicit scheme too heavy from computational standpoint [20]. As a consequence, the convergence of the solution is ensured by keeping very small the integration time steps and the computing time reduced by enabling parallel computing through domain packaging among the workstation processors.

As to the idealization of the structure, different assumptions are made to simplify the modelling and reduce computation costs, resorting to different FE models. The idea is to define and validate several models which can then be adopted to simulate wave–damage interaction so that the most suited model for this kind of application and the specific investigation can be adopted. First of all, it is worth noting that due to the high radius to thickness ratio of the COPV, the wave propagation can be idealized as it happens along a planar media (i.e., thin plate). Generally, the propagation in between a couple of transducers (along the line of sight) can be idealized as a perturbation travelling across a thin plate under specific conditions. Despite COPV being rather complex, when the curvature radius is sufficiently higher than the characteristic dimension of the phenomenon (i.e., characteristic wavelength), this assumption can be accepted. This approach is commonly adopted in literature to characterize the wave propagation according to the material adopted to manufacture the multi-layered structure because returning reasonable results except for the reflections from the borders.

Hence, a multilayered plate is discretized and further assumptions are made. Firstly, a full-3D laminated structure is conceived to idealize the component with highest fidelity possible. Each layer of the structure is discretized as a single lamina, whose material properties are assigned as engineering constants while the local reference system is aligned to the fiber direction. The solid anisotropic medium is idealized through the C3D8R (brick-type) element. As a very small mesh is made necessary by convergence compliance of the explicit integration, the reduced version of the element is preferred to the full formulation to keep lower the computational effort. Indeed, the distance between the element centroid (where the integration is solely carried out) and the nodes (where the displacement information is needed) is so small that does not alter the field of solutions [20].

Secondly, a simplified 2D model is conceived making use of CPE4R elements to idealize the waveguide along the line of sight. Indeed, the detailed 2D plane strain model is extremely useful to accurately discretize the through thickness properties of a multilayered structure while keeping computational cost very low. In addition, this idealization allows to simulate wave propagation under different flaw patterns without resorting to a complex damage discretization. In addition, the model can be easily updated to comply with different propagation directions through rotating material properties. Reduced integration is still adopted to keep time effort lower.

Thirdly, the plate plane is thoroughly discretized as an equivalent single layer (ESL) whose stiffness is the same as that characterizing the section of the three-dimensional plate. In this case, the plate is discretized in detail, while the through thickness behavior is assumed, implementing the first shear deformation theory (FSDT) to derive the displacement functions through the thickness. The displacement approximation is obtained idealizing the composite media through the S4R element and numerically compensated accounting the shear effects [20]. Again, reduced integration is to keep lower computational efforts.

Another significant assumption regards the transducer idealization. Since the dimension thereof is rather limited and usually much smaller than the distance between actuator and sensor, they are discretized as a single node. The actuation is conveyed to the structure forcing the waveform as out of plane displacement. In the same way, the sensing phase is simulated recording the out of plane displacement. The last point of discussion deals with the flaw modelling. Impact induced damage is quite complicated and includes matrix cracks and delamination in between adjacent layers. This latter can be simulated as node detachment in both 3D and 2D approaches efficiently. Instead, although this is still possible with the ESL model duplicating the single layer [22], the shear strain cannot be represented less correctly than introducing a complicated formulation for shear correction factor. Nonetheless, an alternative solution is available and relies on local reduction in material properties through the thickness when defining the layer stacked around the ESL [23].

### 2.4. Signal Processing

#### 2.4.1. Damage Assessment

SHM relying on ultrasonic guided waves as diagnostic method requires the establishment of a dedicated decision framework (to warn the presence of a damage) based on either one or multiple damage metrics. This latter is usually based on a specific feature extracted from the waveform which should be sensitive either to the damage from physics point of view or to any statistical variation of the ultrasonic signal acquired.

When interacting with a delamination-like damage, waves scatter in multiple directions. Along the line of sight, it can be transmitted, reflected, or converted from the discontinuity, showing propagation characteristics different than the incident wave. When crossing damage, the amplitude and the time of arrival of the wave transmitted trough the flaw changes. This is mostly due to the thickness variation of the medium caused by the delamination arising between two adjacent layers and returning a different structural impedance. The damaged zone works as an impedance cage, with part of the energy trapped in between the tips of the delamination [24]. As a consequence, the transmitted wave shows a lower amplitude, which can be caught by a sensor placed beyond the defect. This phenomenon promotes the signal amplitude as a suitable candidate for damage detection, whose metrics in a damage index shape can be defined as:

(2) $$DI_{SA}=\frac{\left|A_{C}-A_{B}\right|}{A_{B}}$$

where the extracted feature *A* is, respectively, evaluated on pristine ($A_{B}$) or currently operating ($A_{C}$) structure.

Likewise, the smaller thickness of the medium reduces the thickness-frequency of the problem and may result in a different arrival time when the wave velocity decreases. This is particularly visible in anti-symmetric mode inspection, showing high dispersivity in the low ultrasound range ([10–80] kHz). Hence, the time of flight among actuator and sensor can be placed as additional feature to gather information about any delamination arising in between them, resulting in the damage index like:

(3) $$DI_{ToF}=\frac{\left|t_{C}-t_{B}\right|}{t_{B}}$$

where *t* is the time of arrival of the transmitted wave. Scattering occurring at the discontinuity can be also caught through waveform energy analysis. As the transmitted and reflected energy varies according to the debonding, any sensor beyond the damage will record less energy travelling through. Meanwhile, any sensors placed before the flaw will register more energy flowing due to the reflected signal back scattered from the defect site. The energy of the signal is included in a damage indicator to detect the defect presence as:

(4) $$DI_{E}=\frac{E\left(S_{C}\left(t\right)\right)-E\left(S_{B}\left(t\right)\right)}{E(S_{B}\left(t\right))}$$

where S(t) is the digitized waveform

Otherwise, damage detection can be achieved in an autonomous way relying on statistical analysis of the waveform. Instead of using a physics based feature, the idea behind this approach is to warn the presence of the damage looking into statistically meaningful variation of the signal using common post processing techniques. Among them, cross-correlation and root mean square deviation evaluated among different signals (i.e.,: baseline and current waveform) are particularly suited to reveal any statistically meaningful variation in the waveform over the time. Comparing the waveforms referred, respectively, to the pristine status and the current damaged condition, the metric warning actual damage is obtained.

Firstly, two signals can be statistically compared computing a classical correlation index, such as the Pearson Correlation Coefficient, ρ, which can be detailed as:

(5) $$\rho =\frac{\left|\sum_{i=1}^{N_{sp}}\left(x_{1,i}-{\bar{x}}_{1}\right)\left(x_{2,i}-{\bar{x}}_{2}\right)\right|}{\sqrt{\sum_{i=1}^{N_{sp}}{\left(x_{1,i}-{\bar{x}}_{1}\right)}^{2}{\left(x_{2,i}-{\bar{x}}_{2}\right)}^{2}}}$$

where $\bar{x}$ represents the average of the samples set. Two perfectly correlated signals returns ρ=1, which is what is expected comparing two inspections when no damage arises. Otherwise, if signals do not match statistically, a smaller correlation coefficient is obtained. That is where damage actually occurs. As such, the detection index is computed as:

(6) $$DI_{CC}=1-\rho$$

Secondly, the damage index approach based on root-mean-square deviation is computed to discern the different states of the structure as follows [25]:

(7) $$DI_{R}=RMSD\left(S_{c}\left(t\right);{\bar{S}}_{b}\left(t\right)\right)$$

In the previous equations S(t) is again the digital signal of the waveform recorded on either the baseline or current condition.

It is worth noting that damage metrics and, as such, damage indicators are affected by any noise in the signals and any environmental disturbances, including electromagnetic coupling and temperature variation. That is the reason why concurrent measurements carried out at the same structural condition and compared through signal processing do not return a null damage indicator, as expected ideally. For this reason, the decision-making framework usually needs a statistical analysis of multiple inspections to assess the noise in an unsupervised mode and chose an opportune threshold of detection.

#### 2.4.2. Damage Localization

Considering a sensor pair used to interrogate the structure, one or more signal responses can be defined to gather any health information along the line of sight. Using a cluster of sensors, a combination of possible interrogation paths is made available and can be exploited to localize the damage. However, this latter diagnosis output is strongly depending upon the efficient reconstruction algorithm. Every information collected along a specific path should be be opportunely weighted and moved to the structure mesh in order to get to a probabilistic distribution of having a damage in a specific point, such as a tomographic representation.

**Figure 3:**
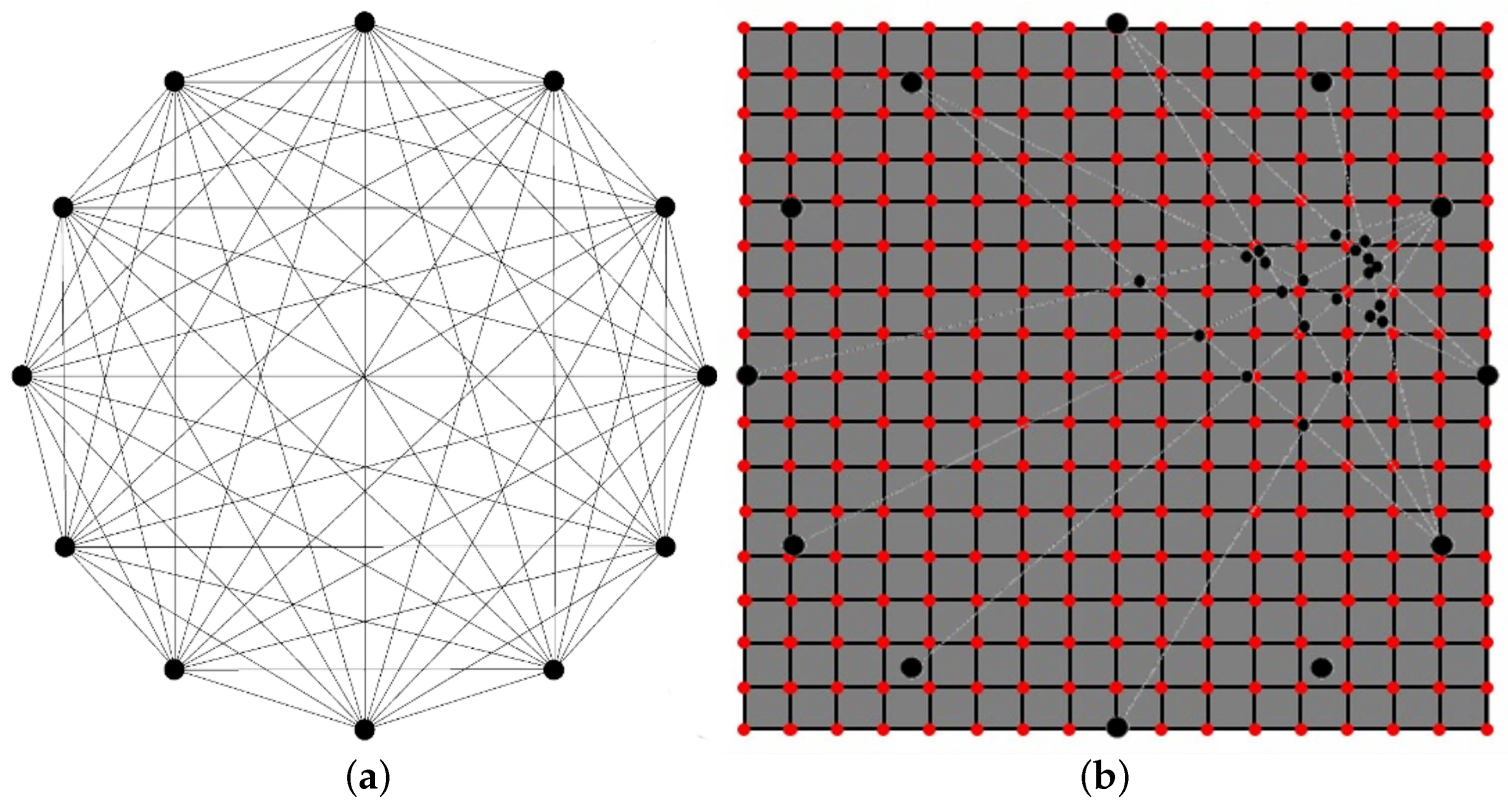
Piezoelectric transducer array and resulting propagation paths where damage indices are calculated (**a**). Structural mesh where damage probability index is derived using probability distribution function (**b**).

Among various reconstruction algorithms, probability based diagnostic imaging is commonly used for guided wave-based SHM as they are easy to implement and do not require many inputs [26,27]. The damage indicators defined by post processing raw signal constitute the signal responses and provide information about damage sensitivity along the direct line of sight in between each pair of transducers. During SHM inspection, transducers are actuated individually, while remaining sensors works as receivers and record wave signals. This procedure returns the network shown in Figure 3a. Structure interrogation is performed multiple times. A first reference state (baseline) is recorded on a known undamaged condition of the structure. Other states (current) are stored via scheduled interrogations. Every time an inspection is carried out, DIs are computed for each transducer pair comparing current state with baseline state. At this point, it is of utmost importance defining how the path related information is distributed and weighted all through the structure. For this reason, a weight distribution function is set up implementing a distance Index (*dI*) for every point of the structure which introduces a decreasing probability according to the distance from the path. The combination of damage responsive indicator and distance related indicator returns a damage probability index (*DPI*) all through the structure as shown in Figure 3b. The i-th path interrogation returns a probability in every point of the structural mesh, *P*, as follows:

(8) $$DPI_{i}\left(P\right)=DI_{i}\cdot dI_{i}\left(P\right)$$

where $dI_{i}$ weights the distance of the point *P* from the i-th path defining a decreasing probability far from it. This mathematical formulation accounts both the damage severity (assessed by the *DI*) estimated along the i-th path and the proximity of a specific point *P* to the i-th path (weighted by *dI*). Finally, the *DPI* at a specific point *P* is evaluated as the normalized cumulative effect returned by every possible path at the point *P*:

(9) $$DPI\left(P\right)=\frac{\sum_{i=1}^{n}DPI_{i}\left(P\right)}{max(DPI)}$$

About the weighting distribution function over the structure, distance indices are computed in three different ways. First, the norm between each structural node and the straight line including both transducers is used to compute the actual distance δ(P) value. Second, this distance is calculated considering both sensors as foci of an ellipse which return iso-probability lines. Finally, the norm between each structural node and the segment connecting transducers is used. The distance index is then evaluated following the Equation (10).

(10) $$dI=\{\begin{array}{c} \frac{\beta -\delta (P)}{\beta -1},\;if\;\delta \left(P\right)\leq \beta \\ \;0\;,\;if\;\delta (P)\geq \beta \end{array}$$

In this formulation, δ(P) is the above mentioned distance and β is the parameter defining the maximum distance up to which the path shows influence. Namely, if the distance is greater than β, the path has no influence on that point and the *DPI* suddenly vanishes. β value is usually calibrated on known condition to improve the reconstruction [28]. Otherwise, it can be optimized following several approaches [29,30].

## 3. Results

This section collects the results obtained from measurements and simulated environments. Firstly, the dispersion characteristics of the $A_{0}$ Lamb wave mode is derived to validate several numerical approaches. Secondly, the simulated environments are used to evaluate the damage indicators to warn the presence of debonding from early stage. Finally, the several signal processing approaches are further developed to localize the damage using the probabilistic reconstruction approach. It is worth noting that the experimental activities are performed mainly to validate the several numerical methods adopted. Indeed, the characterization of the SHM system performed in this paper is not feasible experimentally and requires further simulation to verify that diagnostic parameters (e.g., frequency, wave mode, signal feature, etc.) and damage indices are effective for damage detection. As means of compliance, the dispersion curve of the first fundamental mode is adopted as $A_{0}$ is the most important wave mode excited until 100 kHz, which is the frequency range envisioned for the monitoring approach. This validation is effective because this mode is high dispersive and matching the experimental trend allows to motivate the numerical validation with a very good confidence.

### 3.1. Dispersion Characterization

**Figure 4:**
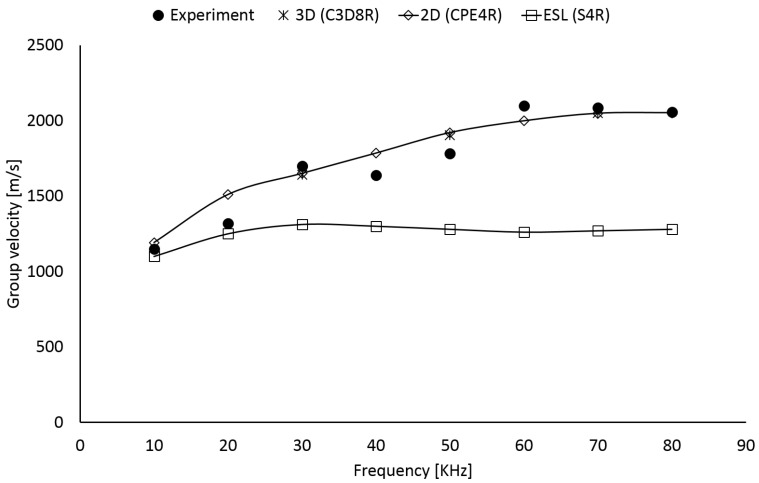
Dispersion curve of $A_{0}$ Lamb wave mode propagating in COPV. Equivalent plate is used to simulate the wave propagation using FE models.

Anti-symmetric fundamental mode of Lamb waves ($A_{0}$) shows severe dispersive behavior in the low ultrasound which results in varying wave velocity versus frequency. The graphical representation of the curve (i.e., dispersion curve) is essential means of validation for FE models. Group velocity can be estimated from the time of flight, as shown in Equation (1), comparing the time of arrival of two wave packets ($t_{1}$ and $t_{2}$), either excited or sensed at two different places using the Short-Time Fourier transform. This calculation is carried out in between 10 and 80 kHz for both measurements and simulations to reconstruct the dispersive trend of the $A_{0}$ mode. Measurements are carried out on the scaled COPV shown in Figure 1b. Instead, numerical investigations are simulated using the equivalent COPV plate idealized with 3D, 2D, and ESL models, respectively. The results are reported in Figure 4.

It is worth noting that the 3D approach well intercepts the dispersion characteristics of the $A_{0}$ mode. This is usually expected due to the high fidelity model conceived. However, the need for discretizing accurately the lamination sequence (resulting in at least one element per ply through the thickness) and being compliant with the convergence constraints given by explicit integration returns a huge number of degrees of freedom. That is to say, the computational cost exponentially increases with structure complexity and it is acceptable only for detailed analysis dedicated to specific investigations.

Instead, the ESL approach based on first shear deformation theory does not predict the dispersion behavior of $A_{0}$ mode unless at very low frequencies. Indeed, when the frequency increases, the wavelength of $A_{0}$ mode decreases and the shear effects start emerging suddenly. This is emphasized by the high thickness of the structure which enables shear effects that cannot be predicted with a first-order shear deformation theory correctly without sorting to a more complex shear correction factor [20]. As suggested by the literature, FSDT is not suited when the slenderness ratio of the plate is too small because Reisnner–Mindlin theory fails to return displacement field through the thickness and even a reasonable shear correction factor does not compensate the error [31]. Despite the benefit in terms of computational efforts gained with ESL approach, material characterization cannot be achieved correctly. An alternative solutions consists in increasing the displacement order (e.g., third order shear deformation theory) to encompass such shear effects. However, this option usually returns computational costs close to those of the 3D approach [32].

Finally, the 2D model returns a reasonable result being able to predict dispersion curve of the $A_{0}$ mode properly all through the frequency range. Indeed, this model allows to discretize in detail the lamination stacking sequence returning the through thickness shear strain arising during wave propagation with high degree of fidelity. In addition, being the discretization limited to the section along the line of sight, the number of degrees of freedom is rather limited even in complex and thick composite structures. As a consequence, a prediction matching is achieved with lower computational cost possible. This enable a versatile tool to analyze a variety of damage patterns in a parametric view. Nonetheless, it is worth noting that this kind of idealization allows simulating wave propagation along one path and requires updating material properties to encompass anisotrpic effect on wave pattern propagation. In addition, the two dimensional boundary does not allow contemplating the three-dimensional in-plane scattering happening at the damage site.

Due to the findings achieved, the 2D model is selected to parametrically investigate the effect of a possible debonding at the metal-CFRP interface towards SHM (i.e., damage metrics versus damage size). Instead, 3D and ESL approaches are adopted further to create data encompassing the full scattering analysis (i.e., damage localization).

### 3.2. Damage Assessment

**Figure 5:**
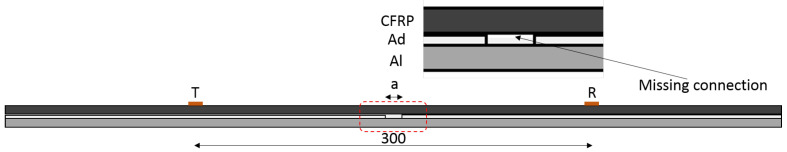
Sketch of the 2D FE model adopted to characterize the metrics vs damage size. The adhesive layer is intentionally enlarged.

Damage is idealized as a perfect debonding between metal and CFRP sides occurring at the interface. Hence, connections among boundary elements are removed at the interface with CFRP to simulate the defect with variable size in between 0 and 25 mm. The sketch of the model adopted and discretized with the 2D FE approach is reported in Figure 5.

**Figure 6:**
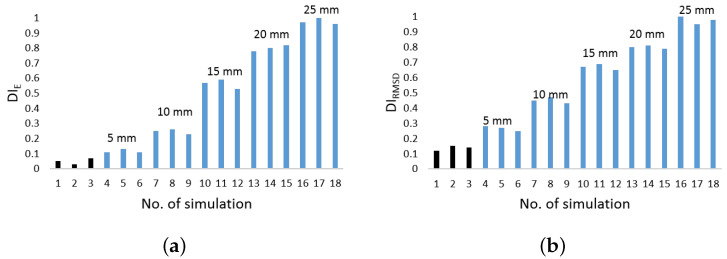
Damage index versus defect size using energy based feature (**a**) and root mean sqare deviation analysis (**b**) carried out at 50 kHz.

Waveforms are recorded in pitch catch mode with actuator and sensor being placed at 300 mm distance and delamination modelled in the middle thereof. Digital data are then post-processed to introduce mathematical noise into the signals and simulate concurrent measurements. This is made necessary to reproduce variability typical of measurements [33]. Typical results obtained computing energy based feature and RMSD analysis are reported in Figure 6a,b, respectively. In both cases the damage indicators are normalized by the maximum value achieved.

The black bars are data obtained when no defect is actually modelled. That is to say, the same structural configuration is interrogated and ultrasonic waves compared together using the same metric as for damage detection. The results obtained are representative of the inherent noise within the signals introduced by mathematical alteration and they are used to calibrate the artificial model on the experimental noisy. Indeed, the DI values obtained are in line with those coming from concurrent measurements. Instead, the blue bars show the damage indicator when such a damage is actually present. Again, the concurrent measurements are simulated via mathematical noise and the data plotted versus the number of simulation and according the defect size.

The findings show that damage detection is possible since defect is early emerging. However, the sensitivity to damage rate and noise level is different, eventually resulting in different reliability. As to this latter aspect, it is worth noting that a quite linear trend of DI versus defect size is found, which is quite important when tackling reliability assessment of the SHM system [34]. However, other influence parameters are crucial in this view, including how defect position affects the detection, which it is not easy handling. Other damage indicators shows a very similar result less than time of flight and signal amplitude. They are both able to warn the presence of the damage. However, the former one does not show a statistically meaningful variability and does not increase linearly. The lack of linearity is still present when evaluating signal amplitude.

### 3.3. Damage Localization

**Figure 7:**
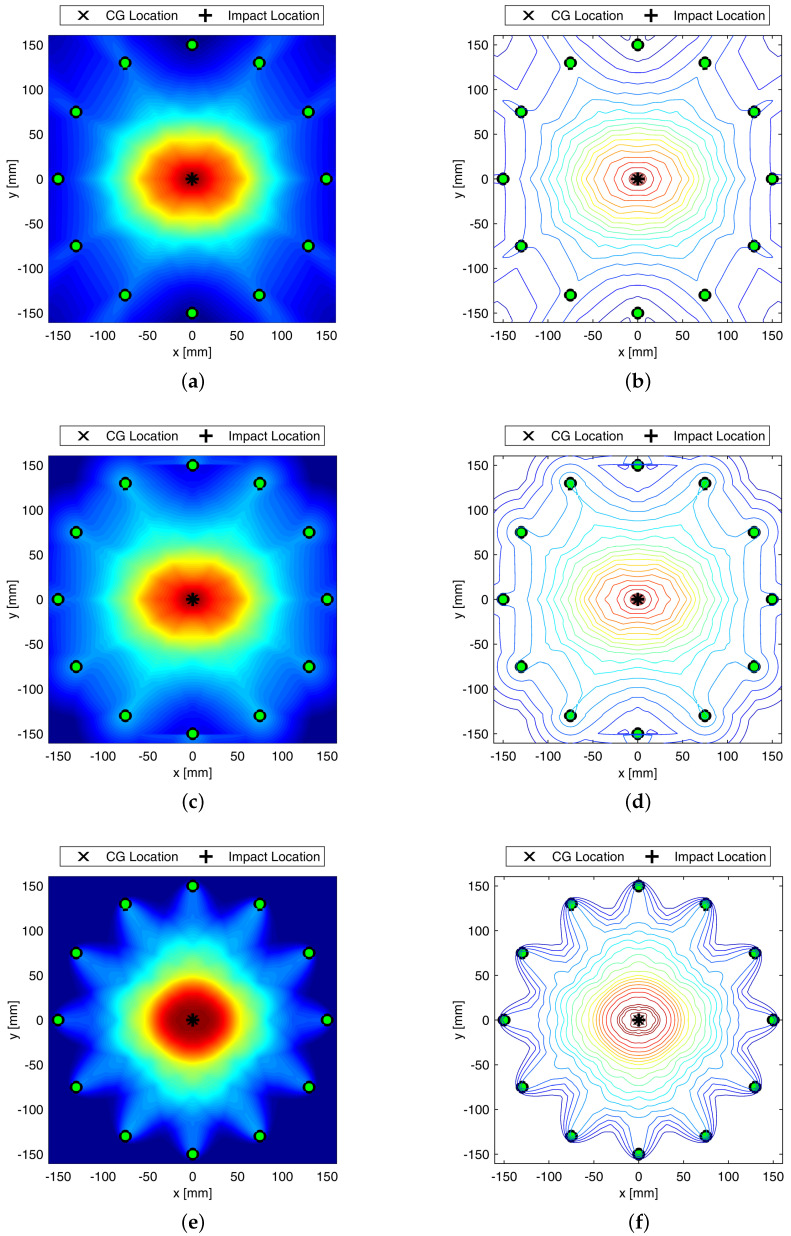
Maps of Damage Probability Index relying on energy based index. Damage map and contour plot using linear (**a**,**b**), modified linear (**c**,**d**), and elliptical (**e**,**f**) weight distribution function. Colorbar in between 0 and 1. SHM carried out at 50 kHz.

This section shows the attempt to localize a damage arising at the aluminum-composite interface in a model assisted way. To enable full in-plane wavefield analysis, the 3D approach is used this time, introducing an actuator and sensor network consisting in 12 transducers uniformly placed all around a 300 mm diameter circle. The damage size is kept constant to 20×20 mm and moved from the center of the plate up to the circumference. Figure 7 shows the detection of centered damage obtained performing different distribution weighting function and optimizing the β parameter. Despite localization is achieved always in a satisfactory way, elliptic weight function returns usually the sharpest reconstruction possible.

**Figure 8:**
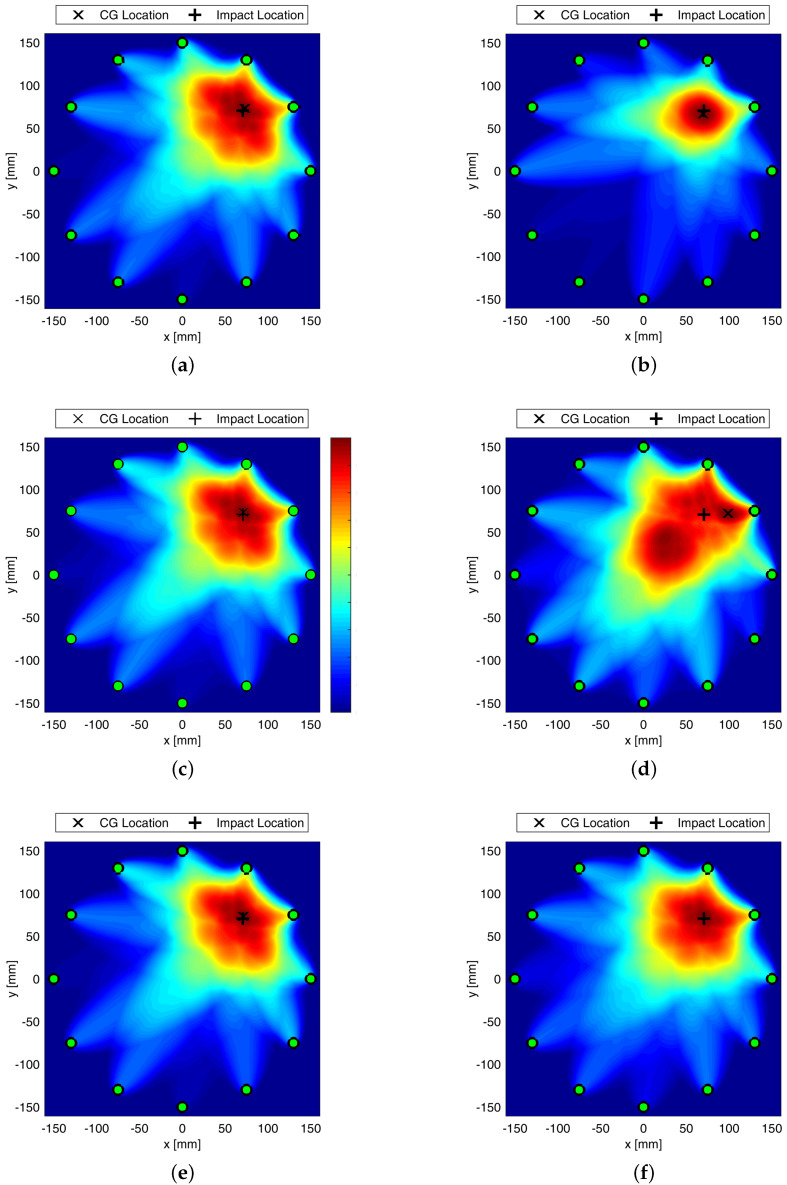
Maps of Damage Probability Index using elliptic weight distribution function relying on energy (**a**), correlation (**b**), RMSD (**c**), time of flight (**d**), signal amplitude (**e**), and fusion (**f**) based damage indicator. Colorbar in between 0 and 1. SHM carried out at 50 kHz.

Since different features are available, the detection was carried out either using a single parameter reconstruction or combining more data to exploit complementary thereof. Figure 8 shows different damage detection results changing the parameter selected for defect assessment. In particular, it is worth noting that time of flight does not return a very good damage reconstruction. Nonetheless, data fusion depicted in Figure 8f shows that error disappears thanks to the features’ complementary.

**Figure 9:**
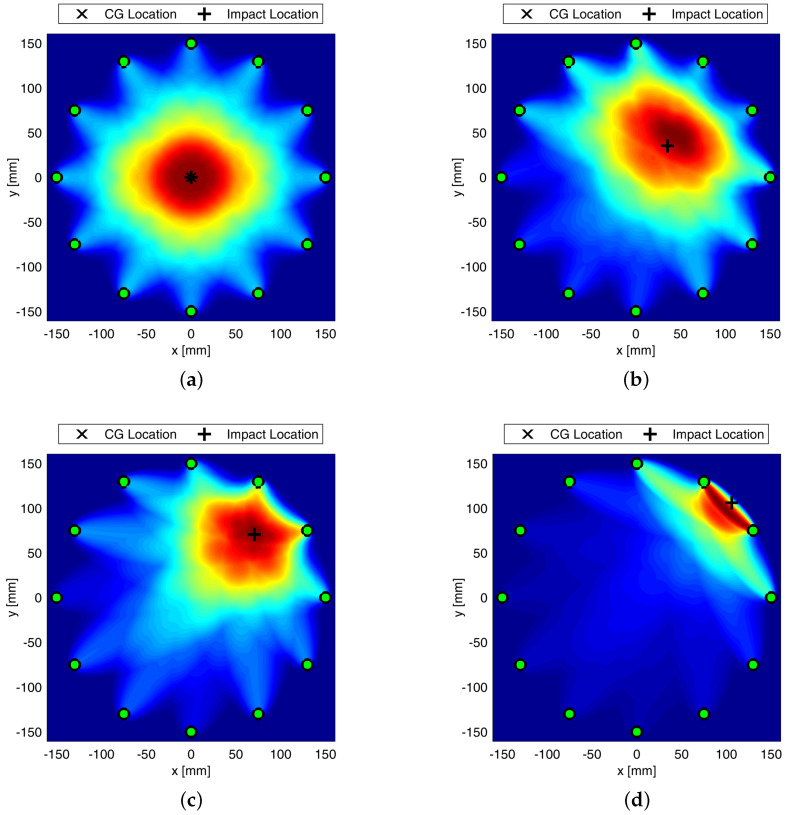
Maps of Damage Probability Index using elliptic weight distribution function and relying on data fusion based damage indicator at 0 mm (**a**), 50 mm (**b**), 100 mm (**c**), and 150 mm (**d**) far away from the center. Colorbar in between 0 and 1. SHM carried out at 50 kHz.

A final investigation regards the position of the damage, which affects the reconstruction quality and, as such, the localization results. However, a satisfactory detection is always achieved while combining all parameters together as depicted in Figure 9.

### 3.4. Discussion

Composite overwrapped pressure vessels are being increasingly used thanks to their inherent advantages in terms of weight to volume ratio and extended life span possible. However, complex manufacturing and composite sensitivity to low velocity impacts still affect the design and threaten safety during lifetime, giving rise to concern. To overcome such a drawback, GW-based SHM is investigated in this paper, showing promising impact even in fields of application undiscovered so far, such as space domain. In particular, the feasibility of Lamb waves is investigated to timely warn damage early emerging during the mission. Propagation behavior is rather complicated by the multilayered media, and supporting simulation is adopted to predict its key characteristics to further investigate and eventually design effective SHM solutions. In this view, this paper shows how different simulation approaches are suited to predict the dispersive behavior of $A_{0}$ propagating and scattering in COPV media. In particular, 2D FE modelling shows promising impact in reducing computational efforts without compromising accuracy. This allowed to look into damage detection sensitivity versus flaw size using a variety of detection metrics, either based on physics phenomena related to wave scattering at the defect or exploiting statistical information gathered within waveform signals. The idea is to rely on different parameters as it happens that each signal feature may be affected by a variety of damage in a different manner. Having more features to look into, enhance the reliability of the system and improves the diagnosis. Here, the different parameters are analyzed against their reliability (Figure 6 shows the effect of damage dimension) and the linear trend of typical features versus flaw size is in accordance with the requirements from [33] for reliability assessment, which can be extended to SHM [34]. Likewise, damage detection and localization potentialities according to the different features are demonstrated in Figure 8, where imaging clearly changes with the feature adopted for damage reconstruction. Finally, the reliability of the system is stressed again against the damage position relying on the multi-parameter approach (i.e., fusion of several information from selected signal features) in Figure 9.

It is worth noting that the characterization of the GW-based SHM system as performed in this paper requires the evaluation of different influencing factors, such as diagnostic parameters (e.g., frequency, wave mode, signal feature, etc.), damage indices versus defect dimension, damage location, and noise. This type of activity is not feasible experimentally and requires further simulation to assess the effect of such factors, as well as the potentiality of the SHM system. Indeed, either a huge number of specimens is necessary or a reversible damage can be introduced (such as sponge, bolts, etc.). Nonetheless, this latter approach relies on a damage idealization and does not reflect the typical impact induce damage. Instead, with a numerical model it is possible to simulate the failure with increasing fidelity, as highlighted in the paper. However, the use of numerical simulation only is not recommended without a prior validation of the numerical model. This is the reason why a first experimental campaign was performed, whose results are used to characterize the dispersion behavior of the anti-symmetric fundamental mode. Hence, once being validated, the idea is to employ the digital twin of the specimen in place of the original specimen. Hence, this is the reason why the numerical analysis is finally extended to explore damage localization possibilities using a three-dimensional model. Information gathered as damage indicators are distributed and weighted all through the structure implementing a probabilistic imaging approach. Results demonstrate that localization is achieved accurately and errors using single feature can be overcome implementing such a data fusion approach to have a reliable defect assessment no matter the position of the damage.

As a final remark, it is worth pointing out that this paper deals with the identification and localization of impact induced damage. Impact is not necessarily due to a long term endurance. Indeed, it is an unforeseen event and results in an abrupt damage through the thickness which needs to be warned and diagnosed promptly. Hence, the main aim of the paper is to demonstrate the efficiency and reliability of a GW-based SHM system. As a more industrial oriented research, a long term testing campaign can be performed to test in a real operating life the condition monitoring approach. However, this would require a devoted research programme for which the feasibility study proposed in this paper is worth being considered as a preliminary statement.

## 4. Concluding Remarks

Composite overwrapped pressure vessels are being increasingly used thanks to their inherent advantages in terms of weight to volume ratio and possible extended life span. However, complex manufacturing and composite sensitivity to low velocity impacts still affect the design and threaten safety during lifetime, giving rise to concern. To overcome such a drawback, SHM is investigated in this paper, showing promising impact even in fields of application undiscovered so far, like space domain. In particular, the feasibility of Lamb waves is demonstrated in this paper to timely warn early damage which emerges during the lifetime. In addition, this paper shows how different simulation approaches are well suited to predict the dispersive behavior of $A_{0}$ propagating and scattering in COPV media. In particular, 2D FE modelling shows promising impact in reducing computational efforts without compromising accuracy. This allows to look into damage detection sensitivity versus flaw size using a variety of detection metrics, either based on physics phenomena related to wave scattering at the defect or exploiting statistical information gathered within waveform signals. Based on these findings, it is possible to asses whether the SHM approach is reliable or not. Finally, extending the numerical analysis to explore damage localization using 3D FE modelling, information gathered as damage indicators can be distributed and weighted all through the structure implementing a probabilistic imaging approach. Results demonstrate that localization can be achieved accurately and errors using single feature can be overcome implementing such a data fusion approach to have a reliable defect assessment no matter the position of the damage. 

## Figures and Tables

**Table 1:** Mechanical properties of aluminum layer (Al), composite material (CFRP), and adhesive layer (Ad).

	*E*_1_ [GPa]	*E*_2-3_ [GPa]	*E*_12-3_ [GPa]	*E*_23_ [GPa]	ν _12-3_	ν _23_	ρ [Kg/m^3^]
Al	71.0	71.0	26.7	26.7	0.33	0.33	2700
CFRP	171.0	8.2	4.1	2.3	0.23	0.42	1520
Ad	2.0	2.0	7.6	7.6	0.30	0.30	1200
